# Is the atherogenic index of plasma a predictor for mortality in ischemic stroke patients?: a retrospective cross-sectional study

**DOI:** 10.1590/1516-3180.2024.0337R1.14072025

**Published:** 2025-09-19

**Authors:** Sefa Tatar, Osman Serhat Tokgöz, Ümmü Gülsüm Selvi

**Affiliations:** I Professor, Department of Cardiology, Meram Faculty of Medicine, Necmettin Erbakan University, Konya, Türkiye.; II Professor, Department of Neurology, Meram Faculty of Medicine, Necmettin Erbakan University, Konya, Türkiye.; III Department of Neurology, Meram Faculty of Medicine, Necmettin Erbakan University, Konya, Türkiye.

**Keywords:** Ischemic stroke, Atherosclerosis, Cholesterol, Triglycerides, Mortality, Ischemic stroke, Cardiovascular diseases, Atherosclerosis, Mortality

## Abstract

**BACKGROUND:**

The atherogenic index of plasma (AIP), derived from the logarithmic transformation of the triglyceride to high-density lipoprotein cholesterol ratio, is frequently used to predict cardiovascular events.

**OBJECTIVE:**

This study aimed to investigate the association between AIP and 1-month mortality in patients with acute ischemic stroke (AIS).

**DESIGN AND SETTING:**

Retrospective study was conducted in Türkiye.

**METHODS:**

In total, 530 AIS patients were enrolled in this study. Clinical, demographic, and laboratory characteristics were recorded within 24 hours of admission. One-month mortality outcomes were analyzed in relation to the AIP of the patients.

**RESULTS:**

Of the 530 patients, 140 patients did not survive during the follow-up period. The mean AIP was 0.50 ± 0.33 in survivors and 0.11 ± 0.27 in the mortality group (P = 0.001). In the receiver operating characteristic analysis, the AIP value of 0.291 had a sensitivity of 74.4%, specificity of 76.4%, positive predictive value of 75.92%, and negative predictive value of 74.9% for mortality. The AIP value above 0.291 had an AUC (area under curve) of 0.829 (95% CI [confidence interval] 0.78–0.88, P = 0.0001). In Cox regression analysis, AIP values below 0.291 (HR 3.962; 95% CI 2.643–5.937) were identified as an independent predictor of mortality. Higher mortality rates were observed in patients with cryptogenic stroke and AIP below 0.291 after stratification by stroke TOAST (P = 0.003).

**CONCLUSIONS:**

Lower AIP is an independent predictor of short-term mortality in AIS patients, surpassing the sensitivity of traditional lipid parameters. This study provides a valuable prognostic tool for clinicians, offering a non-invasive and cost-effective test for a condition associated with substantial mortality and morbidity.

## INTRODUCTION

 Stroke remains a significant health concern owing to its considerable impact on morbidity and mortality.^1^ The early impairment of motor functions, progressive clinical deterioration, and potential for permanent disabilities pose substantial challenges for both patients and their families. Despite advancements in examination and treatment strategies, the prognosis in stroke cases has not shown satisfactory improvement.^2^ Acute ischemic stroke (AIS), comprising 70% of all strokes, is particularly concerning.^3^ Existing classifications for predicting poor prognosis lack clear evidence of superiority, necessitating the exploration of new parameters for assessing prognosis in ischemic stroke patients.^4,5^ Numerous studies have attributed atherosclerosis as a primary cause of ischemic stroke. The evaluation of lipid parameters to predict the atherosclerotic process has become a widely used approach. The atherogenic index of plasma (AIP), calculated as the logarithmic transformation of the triglyceride (TG) to highdensity lipoprotein cholesterol (HDL-C) ratio (log [TG/HDL-C]), is a key parameter for the assessment of dyslipidemia and atherosclerosis. Previous studies have shown that low TG levels are associated with adverse post-stroke conditions. This is generally explained by collateral circulatory dysfunction and low TG levels not responding adequately to increased metabolic stress. Many studies have focused on the positive association between AIP and conditions such as diabetes mellitus, coronary artery disease, and vascular diseases; however, studies on its association with AIS are limited. 

## OBJECTIVE

 This study aimed to establish the AIP as a predictor of 1-month mortality in patients with ischemic stroke. 

## METHODS

### Study population

**Figure 1:**
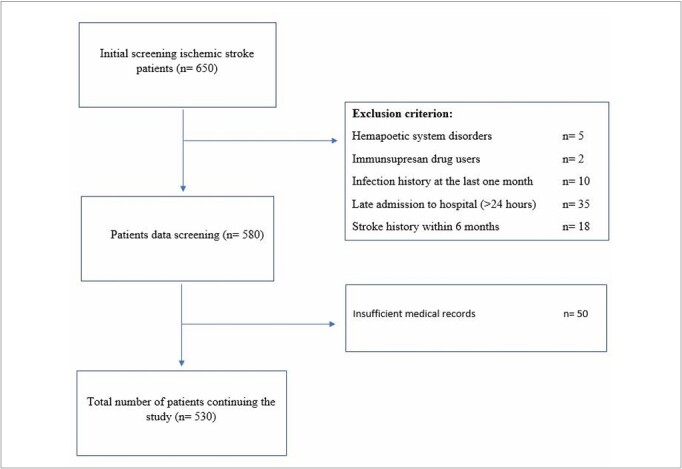
Study flow chart.

 Patients with AIS over the age of 18 years were included in this study. A retrospective analysis was conducted by reviewing the files of patients with AIS between 2012 and 2022. Patients admitted to the hospital with AIS were considered for inclusion, whereas those with strokes occurring after 24 hours were excluded. The exclusion criteria included individuals with hematological diseases, those using immunosuppressive drugs, individuals with a history of malignancy, those with an active infection in the last month, patients with a history of stroke in the last 6 months, those with involvement outside the cortex, and individuals with active diseases directly influencing mortality. The study criteria are shown in **Figure 1**. 

### Study protocol

 A total of 530 patients were included in the study, including 390 surviving and 140 non-surviving patients. The patients were systematically classified based on their demographic, clinical, and laboratory characteristics. Furthermore, the AIP, Modified Rankin classification, and Trial of Org 10172 in Acute Stroke Treatment (TOAST) classification were assessed. Stroke was confirmed using computed tomography and magnetic resonance imaging. Mortality status was verified within 1 month of admission by cross-referencing with the national health screening database (E-Nabız) system. The average acute period of stroke is 15 days. During the chronic period, liquefaction necrosis develops in the brain, which repairs itself and stabilizes. Considering that more than one independent factor will have an impact on mortality in the period after 1 month, 1 month was chosen as the follow-up period. The causes of death of deceased patients were validated through communication with their respective physicians. 

 Patient information, including age, diabetes mellitus, hypertension, history of coronary artery disease, and use of anti-aggregant and anti-coagulant medications, was obtained using an inquiry questionnaire. Comprehensive assessments, incorporating hemogram and biochemical tests, were performed, with specific attention paid to lipid levels. The AIP was calculated based on lipid levels using the formula log (TG/HDL-C). 

### Statistical analysis

 Data analysis was conducted using SPSS software (version 20.0; SPSS Inc., Chicago) and presented as mean ± standard deviation or median (interquartile range). The normality of distribution was assessed using the Kolmogorov–Smirnov test. Independent Student’s t-tests were employed to compare differences between two groups, while the Mann–Whitney U test was used for nonnormally distributed variables. Categorical variable differences were assessed using the chi-square test. 

 Kaplan–Meier survival analysis was performed to explore the association between the median AIP value and mortality. Cox regression analysis was performed to identify predictors of 30-day mortality. The independent variables included in the regression model were age, glucose levels, alanine aminotransferase level, diabetes mellitus, hypertension, coronary arterial disease, WBC count, antiplatelet treatment, and AIP. Receiver operating characteristic (ROC) analysis was performed to determine the specificity and sensitivity of the study. Kaplan–Meier survival estimates for AIP in AIS and survival for AIP were compared according to the median AIP value using the log-rank test. Power analysis was performed using the GPower 3.1.9.7 packet program, which determined the power of a sample size of 530 patients to detect differences in AIP to be 80.5%. 

### Ethical considerations

 This study was approved by the Ethics Committee for Research Involving Human Subjects of the Faculty of Medicine at Necmettin Erbakan University (protocol no. 2024/4781, issued on February 2, 2024). This study adhered to the ethical principles of the Declaration of Helsinki (1964). 

## RESULTS

 In patients with ischemic stroke, the mean age of the surviving group (390 patients) was 66.7 ± 13.3 years, whereas the mean age of the mortality group (140 patients) was significantly higher at 78.5 ± 12.6 years (P value < 0.001). No statistically significant differences were observed between the groups in terms of hypertension, diabetes mellitus, history of coronary artery disease, insulin use, or antiplatelet therapy use. However, variables such as age, glucose level, total cholesterol level, HDL-C level, and WBC count were significantly higher in the mortality group (**Table 1**). 

**Table 1:** Demographic and laboratory findings of surviving and deceased patients

	**Surviving (n = 390)**	**Deceased (n = 140)**	**P value**	**Z value**
Age, y (mean ± SD)	66.7 ± 13.3	78.5 ± 12.6	0.001	
Hypertension (%)	61.3	65.7	NS	
Diabetes mellitus (%)	31	36.4	NS	
CAD (%)	14.1	8.6	NS	
Antiplatelet	5.9	3.6	NS	
Warfarin	4.6	10.7	0.014	5.9*
Oral antidiabetic	12.1	5.7	0.036	5*
Insulin	6.2	11.4	NS	
Glucose (mg/dL), Median (IQR)	113 (75.3)	129 (63.3)	0.008	−4
Creatinine (mg/dL), (mean ± SD)	0.9 ± 0.93	1.02 ± 1.1	NS	
AST	22 ± 18.2	23 ± 11.5	NS	
ALT	19 ± 21.8	15 ± 12.7	NS	
Total cholesterol (mg/dL), (mean ± SD)	173.5 ± 65	162 ± 47.4	0.004	−2.64
Triglyceride (mg/dL), Median (IQR)	116 (79.4)	111.7 (69.1)	NS	
LDL (mg/dL), (mean ± SD)	106 ± 37.1	96 ± 38.6	0.001	−3.51
HDL (mg/dL), (mean ± SD)	37 ± 13	39.1 ± 13.1	0.043	−2.53
Hemoglobin	13.1 ± 1.8	12.9 ± 2.4	NS	
WBC	8.6 ± 4	10.2 ± 4.9	0.002	−3.34
PLT	235 ±1 01.1	235 ± 126.8	NS	
AIP	0.50 ± 0.33	0.11 ± 0.27	0.001	−11.5

CAD, coronary artery disease; SD, standard deviation; AST, aspartate aminotransferase; ALT, alanine aminotransferase; IQR, interquartile range; LDL, low-density lipoprotein; HDL, high-density lipoprotein; NS, not significant; WBC, white blood cell; PLT, platelet; AIP, atherogenic index of plasma

* Chi-square

 There were no significant differences in TG and HDL-C levels between the two groups (116 ± 79.4, 111.7 ± 69.1 mg/dL for TG, 37 ± 13, 39.1 ± 13.1 mg/dl for HDL-C, P > 0.05); however, AIP was significantly lower in the mortality group (0.11 ± 0.27) than in the surviving group (0.50 ± 0.33) (P value = 0.001, Z value = −11.5) (**Table 1**). 

**Figure 2:**
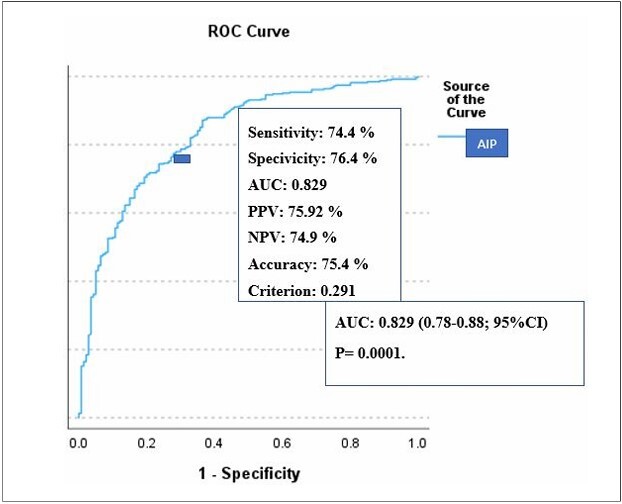
Receiver operating characteristic curve of atherogenic index of plasma prediction of mortality in acute ischemic stroke.

 ROC curve analysis revealed that an AIP value of 0.291 had a 74.4% sensitivity and 76.4% specificity for predicting 1-month mortality. The positive predictive value of AIP for predicting 1-month mortality was 75.92%, with a negative predictive value of 74.9%. The AUC was 0.829 (95% CI 0.78–0.88, P = 0.0001) (**Figure 2**). 

**Figure 3:**
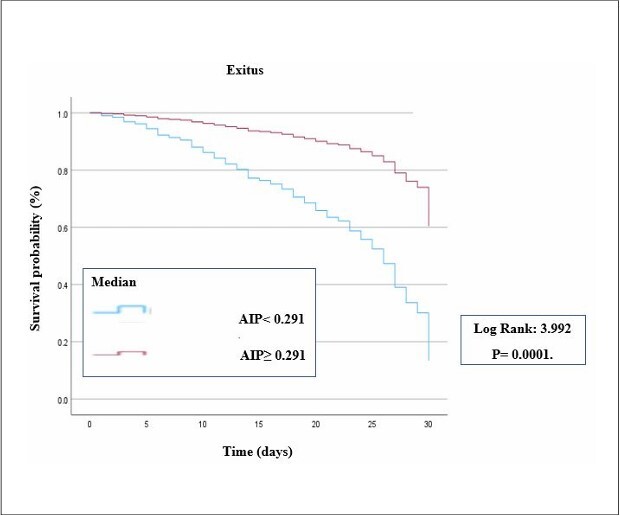
Kaplan–Meier survival estimates for atherogenic index of plasma in acute ischemic stroke and a comparison of survival for AIP according to the median AIP value with long rank test.

 Kaplan–Meier analysis demonstrated a significant difference between the two groups based on the AIP value of 0.291 (**Figure 3**). Age and AIP were identified as independent risk factors for mortality in the Cox regression analysis, with hazard ratios (HR) (95% CI) of 1.048 (1.032–1.064) for age, 3.992 (2.648–6.020) (first step) for AIP, and 3.962 (2.643–5.937) (last step) for AIP (P value < 0.001) (**Table 2**). TGs and HDL-C were not independent predictors in the Cox regression analysis (HR: 1.000 (0.999-1.002), p = 0.581; HR: 1.001 (0.990-1.012), p = 0.889, respectively). 

**Table 2:** Cox regression results for the predictors of mortality

**Variables**		**HR (95% CI)**	**P value**
First step			
	*Age*	1.052 (1.035–1.069)	< 0.001
	*Glucose*	1.002 (0.999–1.005)	0.114
	*ALT*	1.001 (1–1.003)	0.077
	*WBC*	1.027 (0.995–1.059)	0.096
	AIP*	3.992 (2.648–6.02)	< 0.001
	*Hypertension*	1.346 (0.934–1.941)	0.111
	*Diabetes mellitus*	0.817 (0.553–1.206)	0.309
	*CAD*	1.338 (0.737–2.432)	0.339
	*Antiplatelet*	2.136 (0.859–5.317)	0.103
Last step			
	*Age*	1.048 (1.032–1.064)	< 0.001
	AIP*	3.962 (2.643–5.937)	< 0.001

ALT, alanine aminotransferase; WBC, white blood cell; CAD, coronary artery disease; AIP, atherogenic index of plasma

* AIP was included in the regression model as a dichotomous variable as above the median (> 3.962) and below or equal to the median (≤ 3.962)

## DISCUSSION

 This study aimed to explore the role of the AIP in the pathogenesis of stroke and its impact on 1-month mortality in patients with AIS. 

 Atherosclerosis and dyslipidemia are commonly implicated causes of ischemic stroke. Although lipid parameters appear to offer insights into atherosclerosis, the results can be inconclusive. Lipid parameters were not identified as independent predictors in this study. Therefore, these new parameters are needed. Literature reports have highlighted the AIP as a parameter with a higher predictive value than other lipid parameters. Numerous small low-density lipoprotein cholesterol (LDL-C) particles traversing the vascular endothelium interact with glycoproteins in the vascular wall, leading to lipid accumulation.^6^

 Moreover, oxidized LDL-C particles create an environment that facilitates the transformation of monocytes into macrophages with hormonal effects. This process results in foam cell formation and aggregation, triggering the release of large amounts of lipid particles from atherosclerotic plaque and ultimately contributing to stroke. Elevated TG and reduced HDL-C levels are significant risk factors for vascular diseases, regardless of LDL-C levels. Even if LDL-C levels are effectively managed with statin therapy, the vascular risk may persist. Some studies have suggested that the logarithmic ratio of TG to HDL-C has a more predictive effect than considering each parameter individually.^7,8^

 The HDL-C level, which is reported in numerous studies to have protective effects against coronary artery diseases, exerts a significant impact on cardiovascular diseases. However, recent studies have reported conflicting data. Cheng et al.^8^ demonstrated in a study that very high HDL-C levels (> 60 mg/dl) and very low HDL-C levels (< 25 mg/dl) were associated with mortality in patients undergoing percutaneous coronary intervention. Furthermore, evidence suggests that elevated TG levels are a vascular risk factor. The negative impact of these two parameters on lipid metabolism and atherosclerosis is indisputable when considered in isolation. However, the present study indicates that their individual effects on mortality prediction are notably poor. Therefore, the AIP may be deemed as a more sensitive indicator of mortality, given its logarithmic ratio of TG/HDL-C. 

 Wu et al.^9^ established in a study involving 696 patients that the AIP independently predicted the risk of cardiovascular disease. Another study conducted by Garg et al.,^10^ which focused on 267 patients with symptomatic carotid artery stenosis, identified AIP as an independent predictor of carotid vascular risk compared to other lipid parameters. Similarly, a study in China revealed a close correlation between AIP and the severity of cardiovascular disease in patients undergoing coronary angiography.^11^ Won et al.^12^ demonstrated that the AIP exhibits higher sensitivity than traditional risk assessment in detecting an increase in plaque burden in the coronary arteries. Although the study emphasized the pathological nature of a high AIP value in atherosclerosis, it primarily addressed the evaluation of atherosclerosis and the associated stroke frequency rather than mortality. Given this distinction, our study should be regarded as a pilot study exploring the potential differences in mortality pathogenesis compared to atherosclerosis. 

 In the context of coronary artery disease, the AIP is frequently employed as a parameter that indicates the presence of atherosclerosis. Its applicability is more restricted in cerebrovascular and peripheral vascular diseases. However, in recent years, there has been a growing interest in this parameter. Yu et al.^13^ identified AIP as the most crucial determinant of intracranial atherosclerosis. Furthermore, Wang et al.^14^ demonstrated a significant positive linear association between the AIP and ischemic stroke. According to the RICAS study, AIP was positively and linearly correlated with asymptomatic intracranial atherosclerotic stenosis in middle-aged and older patients.^15^ One contributing factor to this association is the disappearance of the anti-atherosclerotic effect of estrogen in the postmenopausal period, coupled with an increase in dyslipidemia with aging. Numerous studies in the literature stated above are vascular risk studies and suggest that a higher AIP may cause cardiological and cerebrovascular diseases. However, such studies have not focused on the outcome and mortality, which is different from our mortality study. 

 Deng et al.^16^ underscored the significance of a lower TG/HDL-C ratio in terms of 3-month mortality in their study, which is consistent with the results of our study. Liu et al.^17^ demonstrated through multivariate logistic regression analysis that AIP independently predicted poor prognosis in patients with AIS, aligning with the outcomes observed in our study. Liu et al.^18^ revealed in their study that a higher AIP was associated with poor prognosis in patients with AIS during the first 3 months, and its predictive value surpassed that of other lipid parameters. A cohort study conducted by Weir et al.^19^ reported that a lower AIP in stroke patients was linked to unfavorable outcomes. The study conducted by Liu et al.^18^ focused on patients with AIS and identified higher AIP as an independent predictor of atherosclerosis occurring in large vessels in stroke mortality. The study stated above was a mortality study; however, it suggested that higher AIP levels were related to mortality, in contrast to our study, which suggested that lower AIP levels were related to mortality. The study cut-off value of AIP in that study ([0.112, HR: 1.84 {95% CI, 1.23–2.53, P = 0.007}]) is different from the cutoff our study ([0.291, HR: 3.962 {95% CI, 2.643–5.937, P < 0.001}]), with a lower sensitivity (59%), and specificity (70%) than our results (sensitivity of 74.4% and specificity of 76.4% in the ROC analysis). Chang et al.8 suggested in a cohort study involving approximately 50 thousand patients that a higher TG-to-HDL-C ratio was associated with positive outcomes. These findings are consistent with those of our study. 

 Çoban et al.^20,21^ highlighted higher TG and TG/HDL-C ratios in young ischemic stroke patients than in older individuals with AIS. The finding was similar to that of our study; the mortality group had mostly older patients compared to the survivors in our results. However, in this study, the AIP was an independent predictor of mortality from age. 

 In our study, stroke of undetermined etiology exhibited higher AIP values according to TOAST classification (**Table 3**). The reason undermined etiology differs from others may be the combination of more than one risk factor; however, the number of samples was relatively small when compared to other groups. Another noteworthy aspect of our study was its focus on mortality rather than prevalence. While numerous studies have explored the correlation between elevated AIP and vascular risk, data regarding the association between AIP and mortality are scarce. This literature supports the notion that lower AIP values, particularly in acute stroke patients undergoing metabolic stress, play a role in meeting the heightened energy demands of TGs, which serve as stored glucose (i.e., low AIP value). The outcomes of these studies are consistent with those of the present study. 

**Table 3:** A comparison of median AIP ratio values among stroke subtypes and the relationship between AIP ratio and survival status for each stroke subtypes

**AIP**				
**Stroke subtype**	**Total**	**Surviving**	**Dead**	**P value**
Major arterial	142 (27.2%)	62 (11.9%)	80 (15.3%)	NS
Cardioembolic	83 (15.9%)	36 (6.9%)	47 (9%)	NS
Minor arterial	115 (22%)	35 (6.7%)	80 (15.3%)	NS
Idiopathic	145 (27.8%)	48 (9.2%)	97 (18.6%)	NS
Cryptogenic	37 (7.1%)	23 (4.4%)	14 (2.7%)	0.003

 Sujatha et al. and Xu et al.^22,23^ found a positive relationship between high AIP and stroke risk. However, the results of our study differ from these results. In our study, we investigated the relationship between AIP and stroke mortality. Several studies have shown that increased TG levels disrupt the vascular bed and increase the risk of stroke. However, there is insufficient evidence suggesting that high TG levels directly affect mortality. Decreased TG levels may reflect malnutrition and increased energy deficits. As a result, the destruction process begins in the body and mediators effective on the endothelium are secreted, which further disrupts the vascular bed. The TG and HDL-C levels in the deceased and living groups were very close and were not statistically significant their differences were not statistically significant. However, as a result of the plasma atherogenicity index calculated with the help of these parameters, mortality can be predicted in ischemic stroke patients. This index is a more sensitive risk predictor than the classical lipid profile. 

 The present study has some limitations. The first is the single-center design of the study, which may affect the generalizability of the findings to broader populations. Furthermore, the age distribution of the patients in our study leans towards advanced age groups. It is crucial to recognize that different results may have been observed in a younger patient cohort. Therefore, caution should be exercised when extrapolating the study outcomes to more diverse populations or varying age groups. Future research involving multiple centers and encompassing a broader age range could provide a more comprehensive understanding of the association between AIP and outcomes in AIS patients. 

## CONCLUSION

 Dyslipidemia is a significant risk factor of ischemic stroke, and atherosclerosis exerts adverse effects on the entire vascular system. Hyperlipidemia and low TG levels are important risk factors for mortality. Numerous studies have investigated various lipid and non-lipid parameters to highlight the unfavorable prognosis of patients with ischemic stroke. However, traditional lipid parameters and other variables do not demonstrate the same level of sensitivity and specificity as AIP in indicating poor prognosis and mortality associated with ischemic stroke. The present study with higher prognostic accuracy and the fact that AIP is a noninvasive and cost-effective test is poised to serve as a valuable resource for clinicians. The findings of this study have the potential to effectively guide clinicians in the future, particularly in the context of stroke, a condition characterized by high mortality and morbidity. 
